# Free and Protected Protease in the Diet of Lactating Jersey Cows: Effects on Performance, Milk Quality, Metabolism, Nutrient Digestibility, Microbiota, and Ruminal Environment

**DOI:** 10.3390/ani16121926

**Published:** 2026-06-22

**Authors:** Maksuel Gatto de Vitt, Andrei Lucas Rebelatto Brunetto, Emeline Pizzolatto de Mello, Tainara Letícia dos Santos, Luisa Nora, Beatriz Danieli, Matheus Wroblescki Silva, Sander Souza Farias, Viviane Cargnin de Lima, Bruna Klein, Camila Ten Kathen Jung, Aniela Pinto Kempka, Gilberto Vilmar Kozloski, Roger Wagner, Miklos Maximiliano Bajay, Aleksandro Schafer da Silva

**Affiliations:** 1 Programa de Pós-Graduação em Zootecnia, Universidade do Estado de Santa Catarina (UDESC), Chapecó 89815-630, SC, Brazil; mak-witt@hotmail.com (M.G.d.V.); andreibrunetto03@gmail.com (A.L.R.B.); emeline@unochapeco.edu.br (E.P.d.M.); tainaraleticia915@gmail.com (T.L.d.S.); miklos.bajay@udesc.br (M.M.B.); 2 Programa de Pós-Graduação em Bioquímica e Biologia Molecular, Universidade do Estado de Santa Catarina, Chapecó 88520-000, SC, Brazil; luisa.nora22@gmail.com; 3 Departamento de Zootecnia, Universidade do Estado de Santa Catarina, Chapecó 89815-630, SC, Brazil; beatriz.danieli@udesc.br (B.D.); matheus.ws@edu.udesc.br (M.W.S.); brunaklein06@yahoo.com.br (B.K.); 4 Programa de Pós-Graduação em Ciência e Tecnologia de Alimentos, Universidade do Estado de Santa Catarina (UDESC), Pinhalzinho 88035-901, SC, Brazil; sander.farias@edu.udesc.br (S.S.F.); aniela.kempka@udesc.br (A.P.K.); 5 Departamento de Ciência e Tecnologia de Alimentos, Universidade Federal da Santa Maria, Santa Maria 97105-900, RS, Brazil; vcargnindelima@gmail.com (V.C.d.L.); rogerwag@gmail.com (R.W.); 6 Departamento de Zootecnia, Universidade Federal de Santa Maria, Santa Maria 97105-900, RS, Brazil; camila.jung@acad.ufsm.br (C.T.K.J.); gilberto.kozloski@ufsm.br (G.V.K.)

**Keywords:** exogenous enzymes, protein utilization, rumen fermentation, dairy cows, microbial modulation

## Abstract

This study evaluated the effects of dietary inclusion of free and protected acid protease on productive performance, milk composition, metabolic profile, nutrient digestibility, and ruminal environment in lactating Jersey cows. Fifteen multiparous cows (67 ± 7.5 days in milk; 27.5 ± 3.5 kg/day) were assigned to a 3 × 3 Latin square (5 squares) design with 21-day periods. Treatments consisted of: control (no enzyme), free protease (4.4 g/day), and protected protease (4.4 g/day). The protected form was developed using alginate-based encapsulation to enhance enzyme stability under ruminal conditions. Protease inclusion did not affect dry matter intake, milk yield, or feed efficiency (*p* > 0.05). However, free protease increased lactation persistency (*p* = 0.05) and improved fat-corrected and energy-corrected milk yields (*p* ≤ 0.02), with intermediate responses observed for protected protease. Milk fat and protein contents were higher in enzyme-fed cows (*p* ≤ 0.05), while other compositional parameters remained unchanged. Apparent crude protein digestibility was greater in cows receiving free protease (*p* = 0.037), with no effects on dry matter or fiber digestibility. Protease intake increased total volatile fatty acid concentrations and major fermentation products (acetate, propionate, and butyrate; *p* ≤ 0.01), indicating enhanced ruminal fermentation. Blood metabolites showed increased total protein and globulin levels in cows fed free protease (*p* ≤ 0.05), suggesting improved protein metabolism. Microbiota analysis revealed no differences in alpha or beta diversity; however, specific microbial taxa and predicted metabolic pathways were modulated by treatments, particularly in post-ruminal compartments. In conclusion, exogenous protease, especially in free form, improved protein utilization and corrected milk production without disrupting microbial stability. These findings highlight the potential of protease as a nutritional strategy to enhance efficiency in dairy systems through targeted modulation of ruminal function and nutrient metabolism.

## Simple Summary

Improving how dairy cows use nutrients in their diet is essential to reduce feeding costs and increase production efficiency. This study evaluated whether adding a protein-digesting enzyme (protease) to the diet of lactating Jersey cows could improve milk production, nutrient use, and animal health. Two forms of the enzyme were tested: one in its free form and another protected by a coating designed to help it resist degradation in the rumen. The results showed that protease intake did not change feed intake or total milk yield. However, cows receiving the enzyme, especially in the free form, produced milk with higher fat and protein content and showed better persistence of lactation. In addition, protein digestibility and indicators of rumen fermentation improved, suggesting more efficient nutrient use. Blood results also indicated better protein metabolism in cows fed the free enzyme. The protected form showed some intermediate effects but did not outperform the free form. Overall, the use of protease can be a promising nutritional strategy to improve milk quality and efficiency in dairy systems without negatively affecting animal health or rumen stability.

## 1. Introduction

The search for improved feed efficiency in dairy cattle production has driven the use of additives capable of optimizing nutrient utilization, particularly protein fractions, which represent one of the highest costs in ruminant diets. In this context, exogenous enzymes have been widely investigated as a strategy to enhance feed digestibility and improve nutrient utilization efficiency in animals [1,2]. Among these, proteases stand out due to their ability to hydrolyze proteins into peptides and amino acids, thereby facilitating their absorption and metabolic use [3]. Acid proteases, such as those derived from Aspergillus niger fermentation, exhibit pepsin-like activity and are particularly effective under low pH conditions [4].

Despite their potential, studies evaluating the isolated use of proteases in ruminants are still limited, and available results are inconsistent. Both in vitro and in vivo studies have shown that protease inclusion can enhance the degradation of plant cell wall components by disrupting protein–fiber linkages present in forages [5,6]. In dairy cows fed high-concentrate diets, protease addition has been associated with improved nutrient digestibility and positive changes in milk composition [7,8]. However, a major challenge affecting enzyme efficacy in ruminants is their stability within the rumen environment, where they may be rapidly degraded by ruminal microbiota, limiting their activity before reaching sites of optimal action [9,10].

To overcome this limitation, enzyme protection strategies have been proposed to modulate the site and rate of enzyme release along the gastrointestinal tract. Technologies such as encapsulation, lipid coating, or polymer-based matrices have been used to protect bioactive compounds from ruminal degradation, enabling their partial release in the rumen or preferential delivery to post-ruminal compartments, such as the abomasum and small intestine. Among encapsulation approaches, systems based on natural polymers, such as alginate, stand out, as they rely on ionic gelation processes to form stable matrices that are responsive to gastrointestinal conditions. In this context, the protection technology employed in the present study, based on the formation of calcium-crosslinked alginate particles, deserves particular attention, as it may directly influence enzyme stability in the rumen and its release profile under post-ruminal conditions. Although this approach has been widely applied to nutrients such as rumen-protected amino acids and lipids, as well as to certain additives including essential oils and fibrolytic enzymes [2,11], studies specifically evaluating protected proteases in ruminants remain scarce.

Evidence from studies using other exogenous enzymes suggests that protection technologies may enhance enzyme stability and efficacy by preventing premature degradation, resulting in more consistent effects on digestibility and animal performance [11,12,13]. Thus, the present study aimed to evaluate the effects of the inclusion of acid protease, in free and protected forms, in the diet of lactating Jersey cows on productive performance, milk composition, animal health, nutrient digestibility, and ruminal environment modulation.

## 2. Materials and Methods

### 2.1. Additive

In this experiment, an acid protease provided by Tectron (Toledo, PR, Brazil) was used. The enzyme is derived from the fermentation of *Aspergillus niger* (source of aspartic protease, pepsin-equivalent activity), with a guaranteed minimum protease activity of 20,000 U/g.

### 2.2. Protease Protection Process and Stability Test

#### 2.2.1. Protection Process

Encapsulation was performed using the external ionic gelation technique, employing sodium alginate as the encapsulating polymer and calcium chloride as the crosslinking agent. Initially, a 3.5% (*w*/*v*) sodium alginate solution was prepared by dissolving the polymer in distilled water under continuous magnetic stirring at elevated temperature (approximately 50 °C) until complete solubilization and formation of a homogeneous solution. The solution was then allowed to cool to below 30 °C and rest to eliminate air bubbles formed during the process. The active material was incorporated into the alginate solution at a proportion of 200 g under gentle stirring to ensure homogeneous distribution within the polymer matrix, avoiding foam formation or compound degradation. Subsequently, the alginate–active mixture was dripped, using a peristaltic pumping system, into an aqueous calcium chloride solution previously prepared and maintained under mild agitation. The distance between the dripping device and the 4% (*w*/*v*) CaCl_2_ solution was standardized to ensure the formation of spheres with uniform morphology. Upon contact with the calcium chloride solution, droplets underwent instantaneous gelation due to ionic interactions between Ca^2+^ ions and alginate carboxyl groups, forming calcium alginate beads. The beads were maintained in the crosslinking solution for 60 min to ensure complete stabilization of the polymer matrix. After gelation, the beads were separated by filtration, washed with distilled water to remove excess unbound calcium ions, and dried in a forced-air oven at 50 °C for 12 h. Illustration of the protected protease in Figure S1.

#### 2.2.2. Stability Test Under Simulated Ruminal Conditions (In Vitro)

To evaluate the stability of the protected protease under simulated ruminal conditions, an in vitro assay was conducted using fresh ruminal fluid. The fluid was collected from two rumen-fistulated Red Angus steers (675 kg) on the day of incubation, filtered through four layers of cheesecloth, and immediately transported to the laboratory. Temperature and pH were recorded, and the fluid was mixed with a previously prepared buffer solution containing macro- and microminerals at a ratio of 1:2 (*v*/*v*). Each incubation flask received 0.5 g of protected protease, corresponding to approximately 20 encapsulated beads, along with 150 mL of buffered ruminal fluid. The flasks were flushed with CO_2_ for 20 s to maintain anaerobic conditions and then incubated in a shaking incubator at 39 °C and 90 rpm for 48 h. The assay was performed in triplicate.

At the end of the incubation period, the integrity of the encapsulated particles was assessed by visual inspection. The beads remained structurally intact, with no evident signs of degradation, indicating high stability of the encapsulation matrix under simulated ruminal conditions. These results suggest that the protection process was effective in preserving the physical integrity of the enzyme during ruminal incubation.

### 2.3. Animals and Housing

The study was conducted at the dairy cattle research unit of the experimental farm of Santa Catarina State University, located in Guatambu, Santa Catarina, Brazil. Fifteen multiparous Jersey cows were used, averaging 67 ± 7.5 days in milk (DIM) and a mean milk yield of 27.5 ± 3.5 kg/day, in a crossover design. Animals were housed in a compost barn system, and milking was performed using an automatic robotic system, DeLaval VMS V300 (DeLaval^®^, Tumba, Sweden), with fully automated processes.

### 2.4. Experimental Design and Diet

The study consisted is a 3 × 3 Latin square (5 squares) with 21-day periods (14 days for adaptation and 7 days for data collection); without a break between periods (stages). All the cows underwent three treatments in a cyclical manner, i.e., Control (CON), with no protease inclusion, Free Protease (FAP), receiving 4.4 g/day of free protease; and Protected Protease (PAP), receiving 4.4 g/day of protected protease. The sequence of treatment assignments described [CON→FAP→PAP, FAP→PAP→CON, PAP→CON→FAP] is a single Latin square sequence.

Cows were fed individually in headlock feeders twice daily (08:00 and 15:00 h), receiving a total of 2 kg/day of concentrate, with or without protease inclusion depending on the treatment. Additionally, to stimulate voluntary visits to the robotic milking system, all cows were offered up to 3 kg/day of pelleted concentrate during milking. The remaining portion of the partial mixed ration (PMR) was provided in collective feeding troughs equipped with an automated system, Intergado (Betim, Brazil), allowing individual animal identification and feed intake measurement. Water was available ad libitum, and all animals were subjected to the same lighting and cooling management. Diets were formulated to meet the nutritional requirements of lactating dairy cows according to National Academies of Sciences Engineering and Medicine [14] (Table S1). The PMR consisted of concentrate, corn silage, and Tifton 85 hay. The chemical composition of the diet is presented in Table 1.

Because the basal diet was identical across treatments and only the protease additive differed, carryover effects were expected to be minimal. However, as no washout period was included between periods, residual effects cannot be completely excluded and could not be formally estimated within the present design.

### 2.5. Zootechnical Data Collection

Productive performance was evaluated through daily milk yield (kg), automatically recorded by the robotic milking system. Lactation persistency was calculated as: (mean milk yield from days 15–21/mean milk yield from days 1–14) × 100.

Based on milk composition, milk yield was standardized using the following corrections:

(a) 4% fat-corrected milk (FCM) was calculated according to National Research Council [15] using the equation: FCM (kg) = 0.4 × milk yield (kg) + 0.15 × milk fat (%) × milk yield (kg);
(b) Protein-corrected milk (PCM) was calculated according to Tyrrell and Reid [16] as: PCM (kg) = milk yield × (0.337 + 0.116 × protein %)/(0.337 + 0.116 × 3.3);
(c) Energy-corrected milk (ECM) was calculated according to Sjaunja et al. [17] as: ECM (kg) = milk yield × (0.327 + 0.116 × fat % + 0.06 × protein %);
(d) Fat- and protein-corrected milk (FPCM), commonly used in European systems and by the Food and Agriculture Organization [18], was calculated according to International Dairy Federation as: FPCM (kg) = milk yield × (0.1226 × fat % + 0.0776 × protein % + 0.249).

Feed intake was primarily recorded using automated feeding systems, where the partial mixed ration (PMR) was available ad libitum throughout the day. Pelleted concentrate intake was also recorded daily via the robotic milking system. Intake from individual feeders was determined by weighing the amount of concentrate offered twice daily (with no refusals, representing total consumption). Total dry matter intake (DMI) was calculated as the sum of PMR intake from the collective feeding system, concentrate intake from individual feeders, and pelleted concentrate supplied via the milking system. Based on these intake and milk production data, feed efficiency was calculated as: feed efficiency = milk yield (kg)/dry matter intake (kg).

### 2.6. Biological Sample Collection

Blood samples were collected on days 1, 14, and 21 of each experimental period. Samples were drawn into tubes containing a clot activator (4 mL) for serum separation and biochemical analyses, and into tubes containing 10% EDTA (4 mL) for complete blood count. Blood samples were stored in a cooled container (±8 °C) and transported to the laboratory. Samples collected in clot activator tubes were centrifuged (QUIMIS, São Paulo, Brazil) for 10 min with a force of 7871 g, and after complete separation, 2 mL of serum were harvested and stored at −20 °C until analysis.

On days 1, 14, and 21 of each experimental period, individual milk samples were collected for compositional analysis and somatic cell count (SCC) using an automatic sampler coupled to the robotic milking system. Approximately 40 mL of milk were collected into vials containing preservative (bronopol) and sent to a certified commercial laboratory (Parleite Laboratory, Curitiba, PR, Brazil). Additionally, on day 21 of each experimental period, milk samples were collected for fatty acid profile analysis.

On day 21 of each experimental period, ruminal fluid and fecal samples were collected and stored using 3M Quick Swabs (3M Company, St. Paul, MN, USA) for transport to the laboratory. Fecal samples were also collected during the last five days of each experimental period (days 17–21) to determine nutrient digestibility coefficients, being obtained directly from the rectal ampulla, individually stored in plastic bags, and immediately frozen (−20 °C). A subsample collected on day 21 was transferred to a commercial biological preservation kit for subsequent fecal microbiota analysis. Additionally, rumen fluid (50 mL) was collected on day 21 of each experimental period, 3 h after feeding, using a silicone esophageal probe coupled to a vacuum pump. These samples were initially refrigerated (±8 °C) and subsequently frozen (−20 °C) for fatty acid profile analysis.

An additional ruminal fluid sample collected on day 21 was transferred to a commercial preservation kit for ruminal microbiota evaluation.

Samples of each dietary ingredient (corn silage, ground concentrate, Tifton hay, and robot-supplied concentrate) were collected during each experimental period and used for chemical composition analysis.

### 2.7. Laboratory Analyses

#### 2.7.1. Feed and Fecal Analyses

Feed and fecal samples were pre-dried in a forced-air oven at 54 °C for 72 h. Subsequently, samples were ground and dried again in a forced-air oven at 105 °C for 24 h to determine dry matter (DM) content. Crude protein (CP) was determined following digestion, distillation, and titration procedures using the Kjeldahl method (method 2001.11), as described by Silva [19]. Ash content was determined by incineration in a muffle furnace at 600 °C for 6 h. Neutral detergent fiber (NDF) was analyzed according to established methodologies [19,20].

#### 2.7.2. Hematology and Biochemistry

Complete blood count was performed using blood samples collected in EDTA tubes and analyzed with an automated hematology analyzer, VET3000 (EQUIP, São Paulo, SP, Brazil). The analyzer provided total counts of leukocytes, lymphocytes, granulocytes, monocytes, and erythrocytes, as well as hemoglobin concentration and hematocrit values.

Serum concentrations of albumin, creatinine, fructosamine, glucose, total protein, urea, cholesterol, triglycerides, gamma-glutamyl transferase (GGT), and aspartate aminotransferase (AST) were measured using commercial kits from Analisa (São Paulo, SP, Brazil) and an automated biochemical analyzer, Zybio EXC-200 (Chongqing, China). Globulin levels were calculated using the equation: globulins = total protein − albumin.

#### 2.7.3. Fatty Acid Profile in Ruminal Fluid

Ruminal fluid samples were thawed at 5 °C and manually shaken for homogenization. Aliquots of 1 mL of the supernatant of ruminal fluid samples were collected in polypropylene microtubes (2 mL) and then centrifuged for 5 min (12,300× *g*). Then, 380 μL of the supernatant was transferred to a new microtube containing 20 μL of formic acid. The mixture was manually shaken and centrifuged for 3 min. After centrifugation, 250 μL of the supernatant of the mixture were collected in another polypropylene tube previously containing 500 μL of isoamyl alcohol solution (833.6 μg mL^−1^ in methanol), used as an internal standard, and were homogenized and centrifuged again. 600 μL of sample was inserted into a 2 mL injection vial. Then, 1 μL of extract was injected into a gas chromatograph equipped with a flame ionization detector (GC-FID; Varian Chrompack CP-3800, Varian Inc., Walnut Creek, CA, USA) and an autosampler (Varian Chrompack CP-8400, Varian Inc., Walnut Creek, CA, USA) in split mode (1:10) at 250 °C. The carrier gas used was hydrogen at a constant pressure of 8 psi. The analytes (acetic, propionic, butyric, valeric, and isovaleric acids) were separated by a CP-Wax 52CB capillary column (60 m × 0.25 mm; 0.25 μm stationary phase thickness). The initial column temperature was set at 50 °C for 1 min, then increased to 120 °C at 25 °C min^−1^, to 230 °C at 10 °C min^−1^, and held at 230 °C for 1 min. The detector temperature was set to 250 °C. Method validation included selectivity, linearity, linear range, repeatability, precision, limit of detection (LOD), and limit of quantification (LOQ) for acetic, propionic, butyric, and isovaleric acids. Linearity was assessed by calculating a regression equation using the least squares method. LOD and LOQ values were obtained by sequential dilutions up to signal-to-noise ratios of 3:1 and 6:1, respectively. Precision was assessed by analyzing the repeatability of four replicated samples. Accuracy was determined by recovering known amounts of the standard substances added to a diluted sample (Table S2). Valeric acid was expressed as the equivalent of isovaleric acid. The results were expressed in mmol^−1^ of each SCFA in the ruminal fluid.

#### 2.7.4. Ruminal Fluid and Fecal Microbiota

Microbial communities were characterized both qualitatively and quantitatively by metagenomic analysis through *16S rRNA* gene sequencing, performed by BPI Biotechnology Research and Innovation.

Total DNA was extracted from 200 mg (wet weight) of samples using the ZR Fungal/Bacterial DNA MiniPrep Kit (Zymo Research, Irvine, CA, USA), following the manufacturer’s instructions. The primers 341F (5′-CCTAYGGGRBGCASCAG-3′) and 806R (5′-GGACTACNNGGGTATCTAAT-3′) were used to amplify the V3–V4 region of the bacterial 16S rRNA gene by polymerase chain reaction (PCR), as described by Klindworth et al. [21]. Libraries were quantified by quantitative PCR using the KAPA Library Quantification Kit (Roche Diagnostics, Tokyo, Japan) following the manufacturer’s recommendations. Samples were normalized to a final concentration of 2 nM and sequenced on an Illumina MiSeq platform using 250 bp paired-end reads.

#### 2.7.5. Determination of Digestibility Coefficients

Feed and fecal samples were weighed in duplicate into polyester bags with 12 µm porosity and incubated in the rumen of a cannulated steer, grazing grass pastures and receiving supplementation with concentrate, for 288 h [22]. After incubation, samples were washed, treated with neutral detergent solution in an autoclave [23] and dried in an oven at 105 °C for 24 h to obtain the iNDF. Digestibility was calculated as: 1 − [(marker in feed/marker in feces) × (nutrient in feces/nutrient in feed)].

#### 2.7.6. Milk Analysis

Milk samples for composition and somatic cell count (SCC) were immediately sent to a commercial laboratory certified by the Brazilian Ministry of Agriculture in Curitiba, Paraná, Brazil. Milk components, including fat, protein, lactose, total solids, solids-not-fat, and urea, were determined by mid-infrared spectrometry according to ISO 9622—IDF Standard 141 [24]. Somatic cell count was determined by flow cytometry following ISO 13366-2—IDF Standard 148-2 [25].

For fatty acid profile analysis, lipid extraction was performed using the method of Bligh and Dyer [26], with modifications. Briefly, 1.5 g of sample was mixed with 0.8 mL of water, 5 mL of methanol, and 2.5 mL of chloroform in a 15 mL polypropylene tube and subjected to mechanical agitation for 30 min. Subsequently, 2.5 mL of chloroform and 1.5% Na_2_SO_4_ solution were added to promote phase separation. The mixture was agitated for 2 min and centrifuged at 2000 rpm for 15 min. Lipids recovered from the chloroform phase were used for fatty acid analysis.

Fatty acid methyl esters (FAME) were prepared by transesterification according to Hartman and Lago [27]. One milliliter of 0.4 M methanolic KOH was added to the extracted lipids, followed by vortex mixing for 1 min. Samples were heated in a boiling water bath for 10 min, then cooled to room temperature. Subsequently, 3 mL of 1 M methanolic H_2_SO_4_ solution were added, followed by vortex mixing prior to chromatographic analysis. FAME analysis was performed using a gas chromatograph, TRACE 1310, equipped with a flame ionization detector (FID) (Thermo Scientific Waltham, MA, USA). One microliter of sample was injected into a split/splitless injector operating in split mode (1:20) at 250 °C. Hydrogen was used as the carrier gas at a constant flow rate of 1.5 mL/min. Separation of FAME was achieved using an RT-2560 column (100 m × 0.25 mm × 0.20 μm film thickness). The oven temperature program was as follows: initial temperature at 100 °C for 5 min, increased to 180 °C at 8 °C/min, then to 210 °C at 4 °C/min, and finally to 250 °C at 20 °C/min, holding for 7 min under isothermal conditions. The detector temperature was maintained at 250 °C. FAME were identified by comparing retention times with those of an authentic standard, FAME Mix 37 (Sigma-Aldrich, St. Louis, MO, USA). Results were expressed as the percentage of each identified fatty acid in the lipid fraction, considering correction factors for FID response and conversion from methyl ester to fatty acid, according to Visentainer and Franco [28].

### 2.8. Statistical Analysis

An experimental design with replicated 3 × 3 Latin square (5 squares) with 21-day periods was used. Initially, a descriptive analysis of the data was performed, followed by assessment of residual normality and homogeneity of variances. As the data met the assumptions of normality, a parametric approach was applied. Data were analyzed using the MIXED procedure of SAS (version 9.4; SAS Institute Inc., Cary, NC, USA), with the Satterthwaite approximation used to determine the denominator degrees of freedom for fixed effects. In the statistical model, treatment, day, period, and the treatment × day interaction were included as fixed effects, while animal was considered a random effect. All measurements obtained on day 1 for each variable were included as covariates in the respective analyses. In addition, initial milk yield was included as a covariate in the model. An autoregressive covariance structure of first order was selected based on the lowest Akaike information criterion (AIC). Least squares mean were compared using the Tukey test, and statistical significance was declared at *p* ≤ 0.05.

Raw paired-end amplicon reads were processed with QIIME 2 [29] (v2026.1) using cutadapt and DADA2 plugins to perform primer removal, denoising and de-replication and produce the Amplicon Sequence Variants (ASVs) table. Taxonomy was assigned with a Naïve Bayes classifier trained on Greengenes2 [30] 2024.09 (trimmed to the V3-V4 region) using the q2-greengenes plugin. Downstream analysis was performed in R (R Core Team 2026) v4.5.2 using phyloseq [31] v1.52 and microeco [32] v1.15.0 packages for ecological analysis.

To assess microbial community structure, alpha diversity was quantified via the Shannon index. Beta diversity patterns were visualized using Principal Coordinate Analysis (PCoA) based on Bray–Curtis dissimilarities. Statistical significance for alpha diversity was determined using the Wilcoxon signed-rank test, while variations in community composition (beta diversity) were assessed using pairwise Permutational Multivariate Analysis of Variance (PERMANOVA).

We performed differential abundance testing with the metagenomeSeq R package [33], which is tailored for sparse marker-gene data. The ASV count data were converted to a MRexperiment object and were normalized using the cumulative sum scaling (CSS) method, which reduces bias from uneven sequencing depth. metagenomeSeq fits a zero-inflated Gaussian (ZIG) model to account for the excess zeros in counts. We then fitted a model with fitFeatureModel, including group as the main variable. Features with adjusted *p* < 0.05 (FDR-corrected) were considered differentially abundant. Because metagenomeSeq explicitly models undersampling and normalization, it often improves sensitivity compared to standard tests.

We predicted metagenomic functions using Tax4Fun2 [34] v1.1.5. Tax4Fun2 uses an annotated 16S reference to map ASVs to KEGG orthologs and pathways. The ASV sequences were used to generate a table of predicted pathway abundances for each sample. These functional profiles were then compared between groups using Linear Discriminant Analysis Effect Size (LEfSe) [35]. LEfSe uses a non-parametric Kruskal–Wallis test to find features with significantly different abundances between groups; pairwise Wilcoxon tests among groups to confirm biological consistency and Linear Discriminant Analysis (LDA) to estimate each feature’s effect size (score threshold of 1.5 to call significant biomarkers).

## 3. Results

### 3.1. Productive Performance

**Figure 1:**
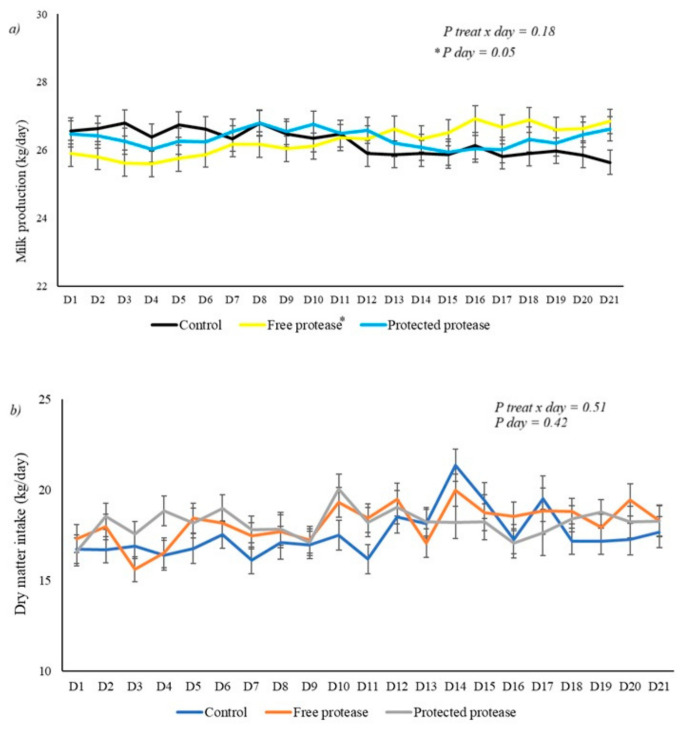
Milk yield (**a**) and dry matter intake (**b**) during the experimental period. The result is presented as the average of three 21-day periods.

No significant differences were observed in milk yield, dry matter intake, or feed efficiency among treatments, although cows receiving protease showed numerically higher milk production during the final days of the experimental period (Figure 1). However, cows receiving free protease showed significantly greater lactation persistency compared to the control group (*p* = 0.05). Regarding corrected milk yield variables, both protease forms resulted in higher fat-corrected milk (*p* = 0.02) and energy-corrected milk (*p* = 0.01) compared to the control group. Protein-corrected milk and fat- and protein-corrected milk were significantly higher in the free protease group compared to the control (*p* < 0.05), whereas the protected protease group showed intermediate values (Table 2).

### 3.2. Milk Composition and Fatty Acid Profile

**Figure 2:**
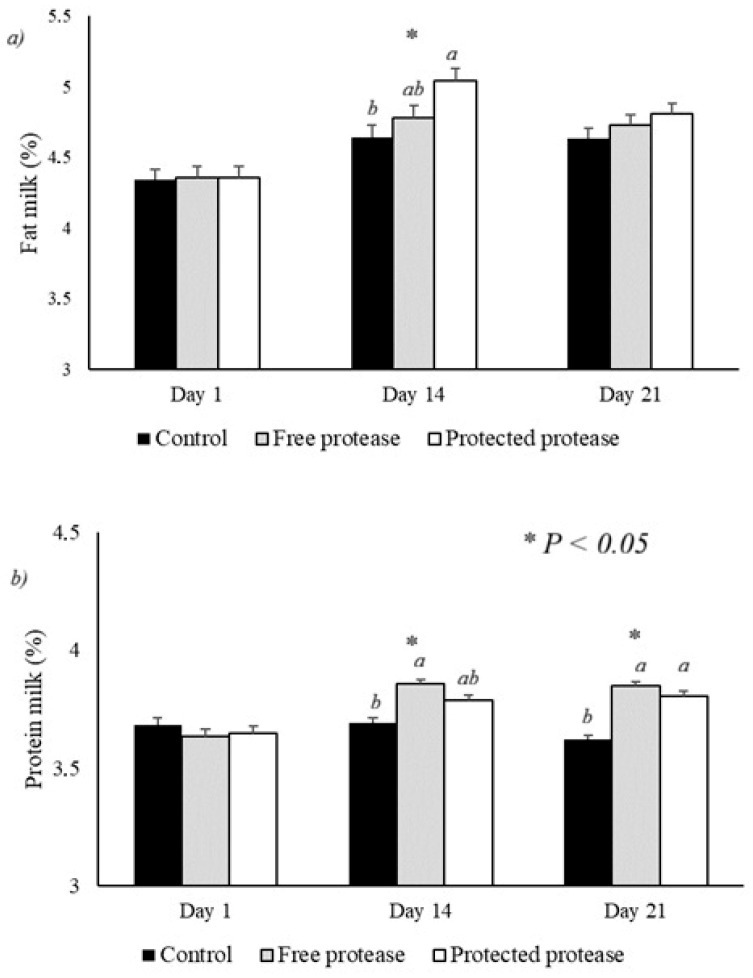
Treatment × day interaction for milk fat (**a**) and protein (**b**) in lactating Jersey cows fed diets with or without free and protected protease. Different letters above the bars indicate differences among groups within each period.

Milk composition data are presented in Table 3. Enzyme inclusion affected milk solids, with increased fat (*p* = 0.05) and protein (*p* = 0.03) contents observed in both FAP and PAP groups, along with a significant treatment × day interaction (*p* < 0.05). On day 14, cows receiving protected protease exhibited the highest milk fat content, whereas on day 21, the free protease group maintained higher protein levels compared to the control (Figure 2). Lactose, total solids, urea, casein, and somatic cell count were not affected by treatments (*p* > 0.05).

The milk fatty acid profile is shown in Table 4. Overall, protease inclusion did not significantly affect most individual fatty acids (*p* > 0.05). However, a treatment effect was observed for oleic acid (C18:1n9c), with lower proportions in the free protease group compared to the other treatments (*p* = 0.05). Similarly, total saturated (SFA), monounsaturated (MUFA), and polyunsaturated fatty acids (PUFA) were not affected (*p* > 0.05). However, total unsaturated fatty acids (UFA) were lower in milk from cows receiving free protease (*p* = 0.05). The UFA/SFA ratio was significantly affected (*p* = 0.01), with lower values observed in the free protease group compared to the control and protected protease groups. Total ω6, ω3 fatty acids, and the ω6/ω3 ratio were not influenced by treatments (*p* > 0.05).

### 3.3. Hematology

Cows receiving free protease showed higher total leukocyte (WBC) and lymphocyte counts compared to the other groups (*p* ≤ 0.05), with the most pronounced effect observed on day 14. Granulocyte and monocyte counts were also affected, with lower values observed in the protected protease group (*p* = 0.05). Red blood cell parameters (erythrocytes, hemoglobin, and hematocrit) and erythrocyte indices (MCV, MCH, and MCHC) were not significantly affected by treatments. These hematological results are presented in Table 5.

### 3.4. Clinical and Metabolic Biochemistry

Clinical and metabolic biochemistry results are presented in Table 6. Total protein concentrations were higher in cows receiving free protease compared to the control group (*p* = 0.05). Globulin concentrations were also affected, with higher values in the free protease group and lower values in the protected protease group (*p* = 0.03). A significant treatment effect was observed for serum urea (*p* = 0.05), along with a treatment × day interaction (*p* = 0.01). On day 21, cows in the FAP and PAP groups maintained more stable urea levels compared to the decrease observed in the control group. No significant differences were observed for albumin, cholesterol, creatinine, fructosamine, glucose, triglycerides, or liver enzymes (AST and GGT).

### 3.5. Apparent Digestibility and Volatile Fatty Acid Profile

**Figure 3:**
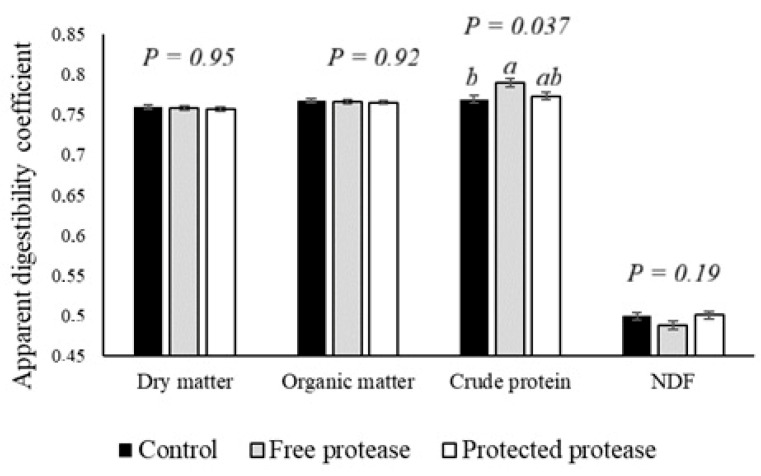
Apparent nutrient digestibility coefficients in cattle fed free and protected protease. Different letters above the bars indicate differences among groups within each variable.

Apparent digestibility coefficients of dry matter, organic matter, crude protein, and neutral detergent fiber (NDF) are presented in Figure 3. No significant differences were observed among treatments for dry matter (*p* = 0.95) or organic matter digestibility (*p* = 0.92). Similarly, NDF digestibility was not affected by protease inclusion (*p* = 0.19). However, a treatment effect was observed for crude protein digestibility (*p* = 0.037), with cows receiving free protease showing higher digestibility compared to the control group, while the protected protease group showed intermediate values and did not differ statistically from the other treatments.

Volatile fatty acid (VFA) concentrations in ruminal fluid are presented in Table 7. Protease inclusion significantly affected several VFA parameters. Acetic acid concentrations were higher in cows receiving both free and protected protease compared to the control (*p* = 0.01), with the highest values observed in the protected protease group. Propionic acid concentrations were also higher in protease-treated groups (*p* = 0.01), as were butyric acid concentrations (*p* = 0.01). Consequently, total VFA concentrations were greater in cows receiving protease (*p* = 0.01). In contrast, isobutyric, isovaleric, and valeric acid concentrations were not affected by treatments (*p* > 0.05). Regarding ruminal protozoa populations, cows receiving free protease showed higher counts compared to the control and protected protease groups (*p* = 0.01).

### 3.6. Ruminal and Fecal Microbiota

**Figure 4:**
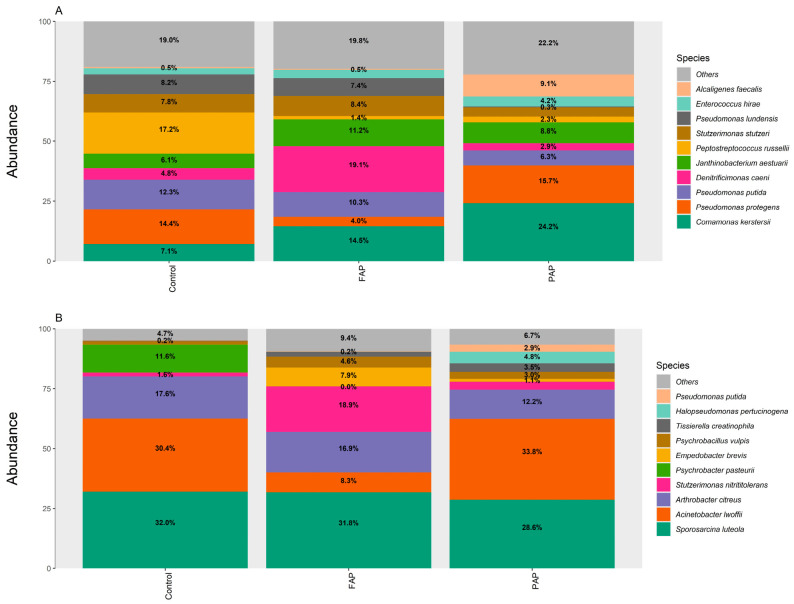
Relative abundance of the top 10 microorganisms in ruminal fluid (**A**) and feces (**B**) of cows fed free acid protease (FAP) and protected acid protease (PAP) compared to the control (no additive).

The relative abundance of the ten most predominant microorganisms identified in ruminal fluid (Figure 4A) and feces (Figure 4B) revealed variations in microbial composition among treatments. In ruminal fluid, the control group showed greater relative diversity among dominant taxa, particularly *Pseudomonas protegens* (14.4%), *Arthrobacter citreus* (12.3%), and *Stutzerimonas stutzeri* (17.2%). The inclusion of free protease (FAP) altered this profile, increasing the abundance of *Comamonas kerstersii* (14.5%) and *Delftinimonas caeni* (19.1%), whereas protected protease (PAP) promoted a higher predominance of *Comamonas kerstersii* (24.2%) and *Pseudomonas protegens* (15.7%). In fecal samples, the microbiota was mainly dominated by *Sporosarcina luteola* and *Acinetobacter lwoffii* across all groups, together accounting for more than 50% of relative abundance. The control group presented 32% of *Sporosarcina luteola* and 30.4% of *Acinetobacter lwoffii*, whereas in the FAP group these values were 31.8% and 8.3%, respectively. In the PAP group, a higher abundance of *Acinetobacter lwoffii* (33.8%) and a lower relative abundance of *Sporosarcina luteola* (28.6%) were observed. Overall, these results indicate that acid protease inclusion, particularly in the protected form, modulated the relative abundance of specific dominant taxa in both ruminal and fecal microbiota, despite considerable inter-individual variation.

**Figure 5:**
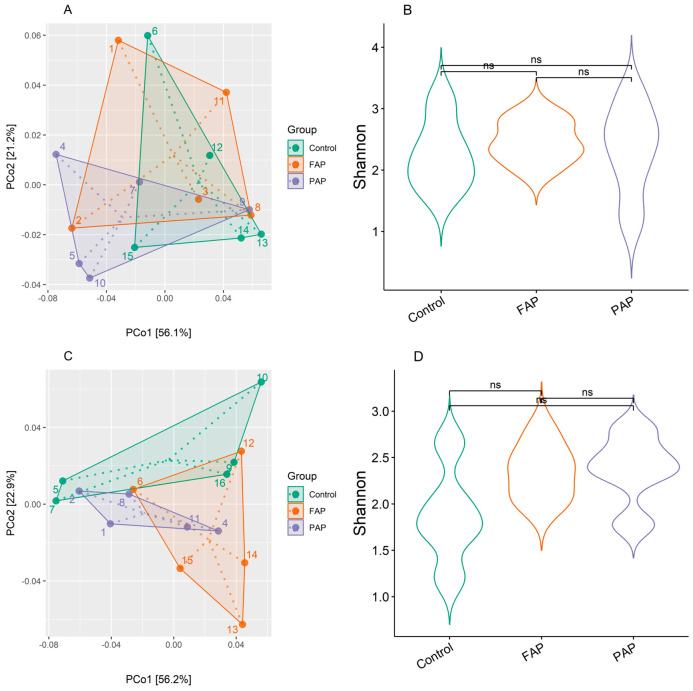
Beta and alpha diversity of microorganisms in ruminal fluid (**A**,**B**) and feces (**C**,**D**) of cows fed free acid protease (FAP) and protected acid protease (PAP) compared to the control (no additive). Note: ns: No significant differences among groups (*p* > 0.05).

Beta diversity analysis based on principal coordinates analysis (PCoA) showed the distribution of microbial communities among treatments for ruminal (Figure 5A) and fecal samples (Figure 5C). In the rumen, the first two axes explained 77.3% of total variation (PCo1 = 56.1% and PCo2 = 21.2%), indicating partial overlap among control, FAP, and PAP groups without clear clustering (PERMANOVA > 0.05 for all pairs). Similarly, in fecal samples (Figure 5C), the first two axes explained 79.1% of total variation (PCo1 = 56.2% and PCo2 = 22.9%), also indicating substantial overlap among treatments. This observation was confirmed by PERMANOVA, which showed no significant differences in ruminal and fecal microbial composition across all pairwise comparisons (*p* > 0.05).

Alpha diversity, assessed by the Shannon index (Figure 5B,D), was not significantly affected by treatments in either ruminal or fecal samples (*p* > 0.05). Together, these findings suggest that protease inclusion, regardless of form (free or protected), did not significantly alter overall microbial diversity in ruminal and fecal communities.

**Figure 6:**
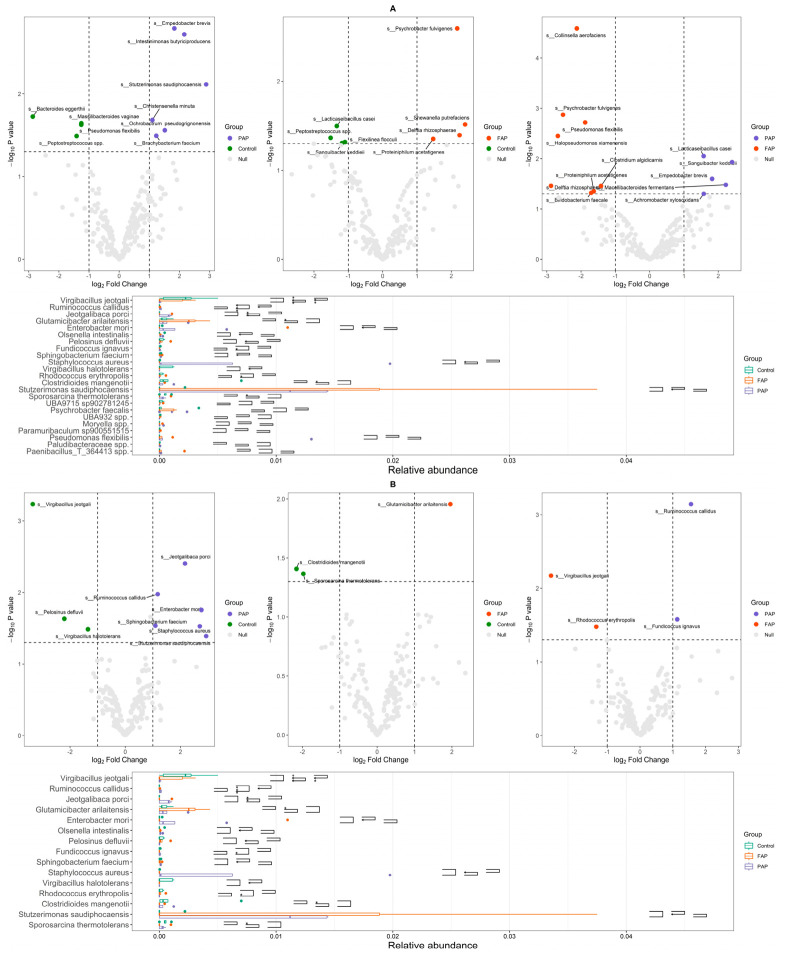
Volcano plots and box plots representing the differential abundance of microbial taxa in the rumen (**A**) and feces (**B**) of lactating Jersey cows supplemented with free protease (FAP), protected protease (PAP), or without enzymatic supplementation (Control). The *X*-axis of the volcano plots represents the magnitude of change in relative abundance (log2FC), while the *Y*-axis represents the level of statistical significance (−log10 adjusted *p*-value). Positive log2FC values indicate higher abundance in the first group of the comparison, whereas negative values indicate higher abundance in the second group. Points are colored according to the group in which the taxon is enriched: blue/purple = PAP; orange/red = FAP; gray = no significant difference (Null). * *p* < 0.05.

Differential abundance analysis of ruminal microbiota (Figure 6A) revealed distinct microbial modulation patterns associated with the type and form of protease. The comparison between FAP and PAP highlighted taxa that differentiated the two enzyme forms, indicating that enzyme protection resulted in a distinct ruminal microbial profile. Taxa with higher abundance in the PAP group included *Collinsella aerofaciens*, *Psychrobacter fulvigenes*, *Halopseudomonas xiamenensis*, and *Pseudomonas flexibilis*. In contrast, taxa enriched in the FAP group included *Lacticaseibacillus casei*, *Sanguibacter keddieii*, *Empedobacter brevis*, and *Macellibacteroides fermentans*. In the comparison between PAP and the control group, significant increases were observed for *Empedobacter brevis*, *Intestinimonas butyriciproducens*, *Stutzerimonas saudiphocaensis*, *Christensenella minuta*, *Brachybacterium faecium*, and *Ochrobactrum pseudogrignonensis*, while *Bacteroides eggerthii*, *Pseudomonas flexibilis*, and *Peptostreptococcus* spp. were less abundant in the PAP group. In the FAP versus control comparison, higher abundances of *Psychrobacter fulvigenes*, *Proteiniphilum acetatigenes*, and *Shewanella putrefaciens* were observed in the FAP group, whereas *Lacticaseibacillus casei*, *Sanguibacter keddieii*, and *Peptostreptococcus* spp. were less abundant.

Fecal microbiota analysis (Figure 6B) reflected cumulative effects of protease inclusion throughout the gastrointestinal tract, revealing significant differences in post-ruminal microbial communities. In the PAP versus control comparison, a marked reduction in transient species of the genus *Virgibacillus* (*V. jeotgali* and *V. halotolerans*) and *Pelosinus defluvii* was observed. Conversely, increased abundances of *Stutzerimonas saudiphocaensis*, as well as core microbiota members such as *Jeotgalibaca porci* and *Ruminococcus callidus*, were detected in the PAP group. In the PAP versus FAP comparison, the PAP group showed higher abundances of *Ruminococcus callidus* and *Fundicoccus ignavus*, while *Virgibacillus jeotgali* and *Rhodococcus erythropolis* were less abundant compared to FAP. In the control versus FAP comparison, lower abundance of *Glutamicibacter arilaitensis* and higher abundances of *Clostridioides mangenotii* and *Sporosarcina thermotolerans* were observed in the FAP group (*p* ≤ 0.05).

Functional prediction of the microbiota revealed differences in metabolic pathways among treatments (Figure 6). In ruminal samples (Figure 6A), linear discriminant analysis (LDA) indicated greater contribution of pathways related to energy metabolism, amino acid biosynthesis, and methane metabolism in the PAP group. Lipid metabolism was more associated with the control group, whereas siderophore biosynthesis from nonribosomal peptides was more prominent in the FAP group. However, when evaluating the relative abundance of these metabolic pathways (Figure 6A), no statistically significant differences were observed among treatments (*p* > 0.05). In fecal samples (Figure 6B), LDA showed that the control group was more associated with pathways related to general metabolism, peptidoglycan biosynthesis, thiamine metabolism, and lipoic acid metabolism. The FAP group showed greater association with functions related to cell motility, flagellar assembly, and carotenoid and terpenoid biosynthesis, whereas the PAP group was more associated with pathways related to valine, leucine, and isoleucine degradation. Unlike ruminal samples, the relative abundance of these functional pathways differed significantly among treatments in fecal samples (Figure 6B).

## 4. Discussion

Research on the use of proteases in ruminants remains limited, and results are often inconsistent [36]. Contrary to our expectations, the inclusion of free and protected protease in the diet did not affect milk yield, a finding also reported by Sucu et al. [37] when using free protease in lactating dairy cows. Similarly, researchers observed that the use of proteolytic enzymes in both low- and high-fiber diets reduced milk production, along with a decrease in dry matter intake [7]. However, in the present study, free protease significantly increased lactation persistency, suggesting a positive modulation of energy or protein balance over time, possibly due to more consistent protein degradation in the rumen. Using an enzyme blend with a higher proportion of protease, our research group previously observed increased milk production only after 17 days, with effects persisting until the end of the experimental period [38]. Therefore, considering that each experimental period lasted 21 days, this duration may have been insufficient to promote significant increases in milk yield, resulting only in numerical differences as shown in Figure 1.

Although milk yield was not affected, higher proportions of fat and protein were observed in milk from cows receiving acid protease (both free and protected forms). These responses resulted in increased protein-, fat-, and energy-corrected milk yields, a phenomenon largely influenced by diet composition [39,40]. The improved protein digestibility observed in this study may explain changes in milk composition, as proteases degrade structural proteins in the plant cell wall, facilitating microbial access to digestible nutrients [13,39]. This likely contributed to the increased availability of precursors for milk solid synthesis. Consistent with this, study reported increased milk fat when protease was included in low-forage diets [7], while other studies using protease combined with additional enzymes reported increased milk protein [38,40].

The intake of exogenous acid protease, either free or protected, did not affect most milk fatty acids, indicating that ruminal lipid metabolism remained relatively stable, as commonly reported for enzymes primarily targeting the protein fraction of the diet [2]. However, the reduction in oleic acid (C18:1n9c), total unsaturated fatty acids (UFA), and the UFA/SFA ratio in the free protease group suggests increased ruminal biohydrogenation. This process converts unsaturated fatty acids into saturated forms, reducing their transfer to milk, and is strongly dependent on microbial activity and rumen conditions [41]. This effect may be associated with enhanced protein degradation in the rumen due to free protease, altering nitrogen availability and favoring microbial populations involved in biohydrogenation [11,42]. In contrast, the absence of such effects in the protected protease group indicates reduced interaction with the rumen environment, likely due to its targeted action in the intestine, thereby preserving the milk fatty acid profile.

Higher levels of total protein and globulins were observed in cows receiving free protease, suggesting improved amino acid availability. Diet is a key factor influencing serum protein levels, and increased protein availability can elevate plasma protein concentrations [43,44]. The behavior of serum urea, which remained stable in enzyme-fed animals at day 21 while declining in the control group, further supports the hypothesis that exogenous protease improved protein degradation and availability. Urea is a well-established indicator of protein metabolism and absorption, and increased protein intake is often associated with elevated blood urea levels [45].

The increase in leukocytes and lymphocytes observed in the free protease group, particularly at day 14, was unexpected. Previous studies suggest that fungal enzymes may act as mild immunomodulators or reflect shifts in gut microbiota that stimulate the immune system [46]. Conversely, the reduction in granulocytes in the protected protease group may indicate a lower inflammatory challenge. According to Zhao et al. [47], ruminal fermentation can induce inflammatory responses in dairy cows, and this effect may be mitigated by enzyme protection, which prevents early ruminal degradation and allows post-ruminal activity. Microencapsulation strategies have been reported as effective in preserving enzymatic activity under ruminal conditions and directing enzyme release to the intestine, thereby enhancing bioavailability and functionality [48]. Another possibility involves the increased availability of peptides and amino acids in the gastrointestinal tract, resulting from protease activity, which may modulate the intestinal microbiota and influence the gut–immune axis. Interactions between the microbiota and the host are recognized to play a central role in regulating immune responses in ruminants [49].

The lack of effect of protease on dry matter, organic matter, and neutral detergent fiber digestibility indicates that the enzyme did not alter overall diet degradation, particularly the fibrous fraction. This is expected, as fiber digestion in the rumen is predominantly driven by fibrolytic microorganisms and is minimally influenced by exogenous proteolytic enzymes. Moreover, previous studies have shown that dry matter digestibility is more responsive to enzymes targeting carbohydrates rather than proteins [8]. In contrast, the increased crude protein digestibility observed in the free protease group demonstrates the effectiveness of the enzyme in enhancing protein hydrolysis within the digestive tract. Exogenous proteases accelerate the breakdown of dietary proteins, increasing the release of peptides and amino acids and improving their availability for digestion and absorption [50]. In ruminants, protein digestibility is closely related to ruminal degradation rates and the fraction escaping to intestinal digestion [51], which explains the stronger effect observed with free protease.

The increase in acetate, propionate, butyrate, and total volatile fatty acids in cows receiving protease indicates enhanced ruminal fermentation activity. Recent studies have shown that exogenous enzymes can increase VFA production by stimulating microbial activity and substrate utilization [52]. This effect is associated with greater availability of degradable nitrogen, resulting from enhanced protein digestion, which supports microbial growth and fermentation efficiency [8]. The simultaneous increase in acetate and propionate suggests that protease did not shift the dominant fermentation pathways but rather increased fermentation intensity. These fatty acids are directly linked to microbial activity and substrate availability in the rumen [53]. The increase in butyrate may be associated with enhanced ruminal metabolic activity, as this fatty acid plays a key role in rumen epithelial development and metabolism [54]. The higher protozoal population observed in the free protease group further supports this interpretation, indicating greater availability of nitrogenous compounds. Protozoa utilize proteins and peptides and play a key role in nitrogen recycling and ruminal fermentation regulation [55]. Overall, these findings suggest that free protease had a greater impact on the rumen environment by increasing protein degradation and fermentation activity, whereas protected protease showed intermediate responses, likely due to limited ruminal activity. This is further supported by the stability test, in which the protected protease remained structurally intact after incubation in ruminal fluid.

When evaluating the relative abundance of microorganisms, we observed numerical effects of the treatment on the 10 main microorganisms found in ruminal fluid, including microorganisms that are more common in the environment. It is also important to note that, although present in the ruminal fluid, they are not as abundant as *Bacteroidetes*, *Fibrobacter*, *Ruminococcus*, and *Prevotella*, which are bacteria abundant in ruminal fluid. Furthermore, strong individual variability in the microbiota of the animals was observed, which we believe is related to the oral composting of these animals, kept in confinement in compost barns, where the animal may ingest bedding, lick objects and walls, as well as lick itself and other animals. In farm animals, analyzing the ruminal microbiota is valid, but the data need to be interpreted with caution.

The results also demonstrate that the intake of free (FAP) or protected (PAP) acid protease modulated specific groups within the ruminal and fecal microbiota of lactating Jersey cows, reflecting changes in fermentable substrate availability, amino acid supply, and ruminal environmental conditions. As shown in Figure 4, protease inclusion in the diet, particularly in its protected form, promoted targeted modulation of the ruminal and fecal microbiota without significantly altering the overall diversity of microbial communities. In the rumen, the most abundant taxa included *Stutzerimonas stutzeri*, *Pseudomonas protegens*, and *Arthrobacter citreus*. Although these taxa did not differ statistically among treatments due to high inter-individual variability, they exhibited relevant numerical variations across groups. These microorganisms are associated with processes such as degradation of complex organic compounds, nitrogen metabolism, and adaptation to different ecological niches within the rumen, potentially influencing fermentation efficiency [56,57,58]. Similarly, in the fecal microbiota, *Sporosarcina luteola* and *Acinetobacter lwoffii* predominated, along with other opportunistic and environmental taxa, reflecting post-ruminal microbial dynamics and cumulative effects along the gastrointestinal tract. Although no statistical differences were observed for these microorganisms, the relative variations among treatments suggest a modulatory response of protease on intestinal microbiota. Overall, the absence of changes in alpha diversity and the high overlap in beta diversity indicate that protease did not promote broad structural changes in the microbial community, but rather adjustments in the relative abundance of specific groups. This behavior is consistent with the literature, which demonstrates that exogenous enzymes primarily act through functional modulation of the microbiota without causing dysbiosis [2,11]. Thus, the results suggest that protease exerts a subtle and targeted effect on the microbial ecosystem, potentially contributing to improved nutrient utilization without compromising ruminal and intestinal microbial stability.

The significant increase in total VFA, acetate, propionate, and butyrate (*p* ≤ 0.01) is directly supported by the enrichment of specific fermentative taxa. In the PAP group, we observed a significant upregulation of *Intestinimonas butyriciproducens* and *Christensenella minuta*. *I. butyriciproducens* is a specialized bacterium known for producing butyrate from the fermentation of lysine and other amino acids. The increased availability of these substrates, liberated by the exogenous protease, likely provided a metabolic niche for this population, directly contributing to the higher ruminal butyrate concentrations. Additionally, the enrichment of *C. minuta* is associated with improved energy efficiency and the production of acetate and butyrate, which are critical precursors for milk fat synthesis and rumen health [59,60]. Thus, the increased availability of peptides and amino acids—likely liberated by the exogenous protease—provided a metabolic niche that favored this population, ultimately enhancing energy supply to the ruminal epithelium [61]. The reduction of Bacteroides *eggerthii* and *Peptostreptococcus* spp. in the PAP group suggests that the exogenous enzyme may have promoted a more synchronized protein hydrolysis, reducing the reliance on ruminal native proteolytic bacteria that often cause excessive deamination [42]. This potentially explains the improved nitrogen utilization and the higher milk protein content observed in treated cows. The greater abundance of *Lacticaseibacillus casei* in the FAP group suggests a fermentative response to increased availability of degraded protein, consistent with the role of exogenous proteases in nitrogen competition within the rumen [13]. The presence of *Shewanella putrefaciens* in this group may indicate changes in ruminal redox potential. The improvement in apparent crude protein digestibility is consistent with the upregulation of *E. brevis*. This taxon is associated with specialized protein degradation. Furthermore, the enrichment of *P. acetatigenes* in the FAP group—a bacterium that utilizes peptides to produce acetate—indicates a shift toward more intensive protein-driven fermentation.

In the fecal microbiome, the results confirm post-ruminal modulation. The reduction of *Virgibacillus jeotgali* in PAP suggests greater residual enzymatic activity in the intestine, consistent with protection against ruminal degradation [62]. The increase in *Ruminococcus callidus* indicates enhanced fibrolytic capacity in the large intestine, contributing to fiber digestibility [63]. The reduction of *Clostridioides mangenotii* in the FAP group may indicate a modulatory effect on potentially opportunistic populations, whereas the increase in *Jeotgalibaca porci* in PAP remains an exploratory finding. Overall, these data indicate that protease, particularly in its protected form, favors microbial populations associated with protein and energy efficiency, with reduced excessive proteolysis and increased short-chain fatty acid production, suggesting improved dietary protein utilization [64,65].

**Figure 7:**
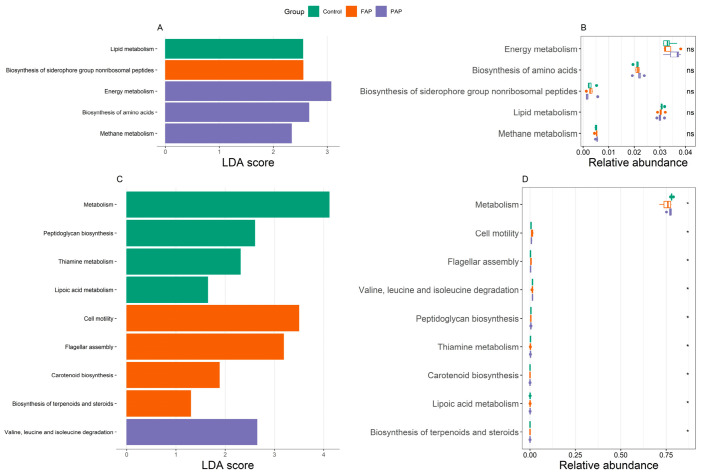
Association between metabolic biomarkers and microorganisms present in ruminal fluid (**A**,**B**) and feces (**C**,**D**) of cows fed free acid protease (FAP) and protected acid protease (PAP) compared to the control (no additive), using LDA scores. Note: * *p* < 0.05; ns—not-significative.

The predicted functional analysis of the microbiota (Figure 7) indicates that protease inclusion promoted specific metabolic modulation, although no significant differences were observed in the relative abundance of major pathways in the ruminal environment, reinforcing the concept of functional redundancy within the ruminal microbiota [57,58]. In the rumen (Figure 7A,B), LDA suggests a greater association of the protected protease group (PAP) with pathways related to energy metabolism, amino acid biosynthesis, and methane metabolism, which may reflect increased availability of nitrogenous substrates and peptides resulting from enzymatic activity, thereby supporting microbial growth and fermentation [56,66]. The association of the ruminal microbiota in the PAP group with pathways related to energy metabolism suggests a more robust fermentative environment. This functional shift aligns with the higher VFA concentrations observed in the PAP group, indicating that the protected protease does not merely provide nitrogen but also enhances the microbial machinery required for efficient energy harvesting from the diet. While exogenous protease increases the availability of peptides, Figure 7 shows a heightened potential for the biosynthesis of amino acids in the rumen. This suggests that the liberated nitrogenous precursors are being effectively utilized for microbial protein synthesis rather than being lost as ammonia. This is further supported by the downregulation of hyper-ammonia-producing bacteria (Figure 6), suggesting a “synchronization” where protein hydrolysis and microbial capture of nitrogen are better balanced. In contrast, the control group showed a greater association with lipid metabolism, while the free protease group (FAP) was associated with the biosynthesis of siderophores from non-ribosomal peptides, a mechanism often linked to microbial competition for iron in highly competitive environments such as the rumen [67,68]. Despite these trends, the absence of statistical differences in the relative abundance of these pathways suggests that the microbiota maintains functional stability in response to the inclusion of exogenous enzymes, as previously demonstrated in studies with enzymatic additives in ruminants [2,11]. In contrast, in fecal samples (Figure 7C,D), more pronounced differences were observed among treatments, indicating that the effects of protease may be more evident in the post-ruminal environment. The control group was more associated with pathways related to basal metabolism and biosynthesis of cellular structures such as peptidoglycan, while the FAP group showed greater association with functions related to cell motility and flagellar assembly, which may indicate greater ecological plasticity and microbial adaptation [69]. The PAP group, in turn, showed greater association with the degradation of branched-chain amino acids (valine, leucine, and isoleucine), suggesting increased flow of undegraded nitrogenous compounds from the rumen to the intestine, consistent with the hypothesis of enzyme protection and ruminal bypass [70,71]. This is a critical metabolic marker indicating that the protected protease likely maintained activity into the lower digestive tract, increasing the flow of bypass protein and peptides. The degradation of these amino acids in the hindgut produces branched-chain fatty acids, which can be utilized by distal microbiota or the host, reflecting a shift in the primary site of proteolytic activity compared to the FAP group. Conversely, the fecal microbiota of the FAP group was more associated with Starch and Sucrose Metabolism. This suggests that without the protection of the enzyme, proteolytic activity was largely exhausted in the rumen, leaving the distal microbiota to focus on residual carbohydrates. This contrast highlights the efficacy of the encapsulation technology in delivering enzymatic activity throughout the gastrointestinal tract. Overall, these findings indicate that while protease intake does not disrupt the core microbial architecture of the rumen, this stability is a desirable outcome in nutritional interventions. It suggests that the observed improvements in milk production and VFA profiles stem from the fine-tuning of specific metabolic functions—likely driven by functional redundancy—rather than from potentially harmful ecological shifts or dysbiosis. Furthermore, the ability of protease to functionally modulate microbial communities, particularly in post-ruminal compartments, has direct and positive implications for nutrient metabolism and feed efficiency.

In general, enzyme protection was not able to enhance protein digestibility, thereby rejecting our initial hypothesis. In the in vitro rumen assay, no apparent changes in capsule weight or morphology were observed. However, post-ruminal conditions were not experimentally evaluated, and it is therefore not possible to ensure the complete release of the encapsulated protease, which represents a limitation of this study. Calcium alginate encapsulation is widely employed as a strategy to protect bioactive compounds from premature degradation in the ruminal environment and to modulate their release along the gastrointestinal tract, due to its biocompatibility and ability to form ionically crosslinked gels [72]. The release behavior of alginate-based systems is strongly dependent on environmental conditions, particularly pH and ionic composition, including the presence of competing ions such as phosphates [73]. Under strongly acidic conditions, alginate matrices undergo protonation of carboxyl groups, leading to contraction of the polymer network and reduced permeability, which limits the diffusion and release of encapsulated compounds [74]. Although these conditions contribute to relative structural stability, they may impair the effective release of encapsulated enzymes. In contrast, at near-neutral pH values—especially in the presence of phosphate ions and digestive secretions such as pancreatic fluid—ion exchange processes involving Ca^2+^ displacement and increased polymer ionization promote matrix swelling and structural weakening, thereby facilitating the release of encapsulated compounds [72,73]. In ruminants, this physicochemical behavior becomes particularly relevant due to the progressive increase in pH along the gastrointestinal tract, from acidic conditions in the abomasum (pH ~2.5–3.0) to near-neutral conditions in the small intestine (pH ~5.5–6.5) [75,76]. Therefore, the release of encapsulated enzymes in alginate-based systems is unlikely to occur predominantly under acidic conditions, but rather during post-abomasal stages, where the environment becomes more favorable for matrix disintegration. These findings underscore the importance of considering the entire gastrointestinal pH gradient when evaluating encapsulation efficiency and functional delivery of enzymes in ruminant nutrition systems. Moreover, as the release profile is formulation-dependent, experimental validation under simulated gastrointestinal conditions is essential to confirm both the site and extent of enzyme release.

A limitation of the present study is the relatively small sample size, which may have reduced statistical power to detect subtle treatment effects, particularly in microbiome analyses where inter-individual variability is inherently high. This may limit the ability to detect biologically relevant differences among treatments. In addition, although the encapsulation process demonstrated high structural stability under simulated ruminal conditions, the release kinetics of the encapsulated protease under post-ruminal conditions were not experimentally verified. Furthermore, no direct measurements of enzyme activity were performed in post-ruminal compartments. Our hypothesis is that protecting the protease could improve its utilization and enhance its effects, since its action would occur in the intestine. However, this did not happen when we considered protein digestibility; this may be related to the lack of certainty about the enzyme’s site of activity in the gastrointestinal tract, as well as a lack of knowledge about its release kinetics. Therefore, conclusions regarding the site-specific activity of the protected enzyme should be interpreted with caution.

## 5. Conclusions

In summary, the inclusion of acid protease in the diet of lactating Jersey cows, particularly when provided in free form, enhanced productive efficiency by improving corrected milk yields and crude protein digestibility, while also promoting favorable shifts in metabolic indicators and ruminal fermentation end-products. Although no major changes were observed in overall feed intake, milk yield, or global microbial diversity, the enzyme modulated specific microbial taxa and functional pathways, suggesting a targeted effect on nutrient utilization rather than broad alterations of the rumen ecosystem. The protected form suggests a potential for modulating post-ruminal metabolism, reinforcing the hypothesis of partial rumen bypass; however, its effects were generally less pronounced than the free enzyme under the conditions evaluated. Collectively, these findings advance current knowledge by demonstrating that exogenous protease, even without drastically reshaping the microbiome, can fine-tune microbial functionality and improve protein use efficiency in dairy systems, representing a promising nutritional strategy to optimize performance and metabolic responses in lactating cows.

## Figures and Tables

**Table 1:** Composition of the diet fed to animals during the experimental period.

Item	PMR	CON Concentrate	FAP Concentrate	PAP Concentrate	Robot Concentrate
Dry matter (DM), %	45.31	87.31	87.96	87.50	89.52
Ash, % of DM	5.85	9.19	9.53	9.50	4.36
Crude protein, % of DM	14.82	25.66	25.95	25.58	18.74
NDF, % of DM	30.81	22.69	24.37	23.95	17.48

PMR: Partial Mixed Ration; CON: Control; FAP: Free Protease; PAP: Protected Protease.

**Table 2:** Productive performance, feed intake, and feed efficiency of cows fed free and protected protease.

Variables	Control	Free Protease	Protected Protease	SEM	*p*-Treat ^7^	*p*-Treat × Day	*p*-Period
Milk production, kg/day					0.09	0.18	0.82
d1 to 14	26.42	26.12	26.45	0.40			
d15 to 21	25.88	26.80	26.27	0.40			
Dry matter consumption, kg/day	17.94	18.67	18.10	0.65	0.74	0.51	0.91
Feed efficiency, kg/kg ^5^	1.44	1.44	1.45	0.07	0.92	0.87	0.96
Persistence of lactation, % ^6^	97.9 ^b^	102.6 ^a^	99.3 ^ab^	0.62	0.05	NE	0.68
FCM, kg/day ^1^	28.3 ^b^	29.8 ^a^	29.9 ^a^	0.35	0.02	NE	0.71
PCM, kg/day ^2^	27.5 ^b^	29.5 ^a^	28.6 ^ab^	0.33	0.001	NE	0.73
FPCM, kg/day ^4^	28.4 ^b^	29.4 ^a^	28.9 ^ab^	0.27	0.05	NE	0.65
ECM, kg/day ^3^	28.0 ^b^	29.7 ^a^	29.5 ^a^	0.27	0.01	NE	0.79

Note ^1^: Fat-corrected milk (FCM) was estimated by the equation proposed by the NRC [15]: FCM = 0.4 × (kg of milk produced) + 0.15 × (% fat) × (kg of milk produced). Note ^2^. Production corrected for 3.3% protein according to Tyrrell & Reid [16]: Protein-Corrected Milk (PCM) = Milk production × (0.337 + 0.116 × % Protein)/(0.337 + 0.116 × 3.3). Note ^3^. Milk production corrected for energy [17]: ECM (kg) = Milk (kg) × (0.327 + 0.116 × % Fat + 0.06 × % Protein). Note ^4^. Fat and Protein Corrected Milk (FPCM), used by European systems and the FAO to standardize milk at 4.0% fat and 3.3% protein using the equation [18]: FPCM (kg) = Milk (kg) × (0.1226 × % Fat + 0.0776 × % Protein + 0.249). Note ^5^. Feed efficiency = production (kg)/consumption (kg). Note ^6^: Lactation persistence was defined by the equation: (average milk production d15–21/average milk production d1–14) × 100. Note ^7^: Treatment effect when *p* < 0.05, illustrated by different letters on the same line. NE: Not-evaluated.

**Table 3:** Milk composition of lactating Jersey cows fed diets with or without free and protected protease.

Variables	Control	Free Protease	Protected Protease	SEM	*p*-Treat ^1^	*p*-Treat × Day	*p*-Period
Fat (g/100 g)	4.64 ^b^	4.75 ^ab^	4.93 ^a^	0.09	0.05	0.03	0.94
Protein (g/100 g)	3.66 ^b^	3.85 ^a^	3.80 ^a^	0.03	0.03	0.01	0.62
Lactose (g/100 g)	4.68	4.66	4.68	0.02	0.92	0.94	0.92
Solid total (g/100 g)	14.0	14.5	14.3	0.11	0.39	0.15	0.81
Urea (mg/dL)	18.1	17.7	18.0	0.47	0.94	0.91	0.95
SCC (×1000/mL)	126	106	120	17.3	0.91	0.85	0.55
Casein (g/100 g)	2.97	3.05	3.03	0.02	0.76	0.67	0.98

Note ^1^: Treatment effect when *p* < 0.05, illustrated by different letters on the same line.

**Table 4:** Fatty acid profile in milk of cows fed diets with or without free and protected protease.

Variables	Control	Free Protease	Protected Protease	SEM	*p*-Treat ^1^	*p*-Period
C4:0 (Butyric)	0.37	0.38	0.41	0.03	0.54	0.97
C6:0 (Caproic)	0.67	0.67	0.66	0.03	0.97	0.88
C8:0 (Caprylic)	0.73	0.74	0.68	0.03	0.85	0.84
C10:0 (Capric)	2.56	2.66	2.49	0.07	0.64	0.91
C11:0 (Undecanoic)	0.23	0.24	0.22	0.01	0.81	0.89
C12:0 (Lauric)	3.69	3.90	3.65	0.11	0.78	0.76
C13:0 (Tridecanoic)	0.16	0.17	0.16	0.01	0.96	0.92
C14:0 (Myristic)	12.6	13.1	12.6	0.22	0.09	0.95
C14:1 (Myristoleic)	0.59	0.60	0.59	0.02	0.98	0.87
C15:0 (Pentadecanoic)	1.21	1.24	1.18	0.04	0.72	0.97
C16:0 (Palmitic)	41.0	41.8	41.2	0.43	0.95	0.95
C16:1 (Palmitoleic)	1.17	1.14	1.21	0.05	0.24	0.88
C17:0 (Heptadecanoic)	0.43	0.39	0.41	0.02	0.79	0.91
C17:1 (cis-10-Heptadecenoic)	0.12	0.11	0.12	0.00	0.96	0.86
C18:0 (Stearic)	13.7	13.3	13.7	0.26	0.67	0.90
C18:1n9t (Elaidic)	1.44	1.24	1.40	0.05	0.10	0.76
C18:1n9c (Oleic)	16.5 ^a^	15.6 ^b^	16.6 ^a^	0.27	0.05	0.54
C18:2n6c (Linoleic)	1.71	1.71	1.70	0.05	0.97	0.98
C20:0 (Arachidic)	0.17	0.16	0.17	0.00	0.98	0.93
C18:3n6 (Linolenic)	0.02	0.02	0.02	0.00	0.99	0.99
C20:1n9 (cis-11-Eicosenoic)	0.05	0.04	0.05	0.00	0.98	0.99
C18:3n3 (a-Linolenic)	0.14	0.13	0.13	0.00	0.96	0.98
C21:0 (Henicosanoic)	0.29	0.26	0.27	0.01	0.93	0.94
C20:2 (cis-11,14-Eicosadienoic)	0.03	0.03	0.03	0.00	0.99	0.99
C22:0 (Behenic)	0.06	0.06	0.06	0.00	0.99	0.99
C20:3n6 (cis-8,11,14-Eicosatrienoic)	0.07	0.08	0.08	0.00	0.98	0.98
C22:1n9 (Erucic)	0.02	0.03	0.02	0.01	0.97	0.99
C20:4n6 (Arachidonic)	0.04	0.04	0.03	0.00	0.98	0.99
C22:2 (cis-13,16-Docosadienoic)	0.01	0.01	0.01	0.00	0.99	0.99
C24:0 (Lignoceric)	0.04	0.04	0.03	0.00	0.97	0.99
C20:5n3 (cis-5,8,11,14,17-Eicosapentaenoic)	0.01	0.01	0.01	0.00	0.99	0.99
Other variables						
∑ Saturated fatty acids (SFA)	78.0	79.1	77.9	0.34	0.06	0.90
∑ Unsaturated fatty acids (UFA)	21.9 ^a^	20.8 ^b^	22.0 ^a^	0.33	0.05	0.78
∑ Monounsaturated fatty acids (MUFA)	19.9	18.8	19.9	0.38	0.29	0.92
∑ Polyunsaturated fatty acids (PUFA)	2.03	2.03	2.01	0.05	0.94	0.96
UFA/SFA	0.28 ^a^	0.26 ^b^	0.28 ^a^	0.01	0.01	0.75
∑ ω6	1.85	1.85	1.83	0.05	0.95	0.95
∑ ω3	0.15	0.14	0.14	0.00	0.96	0.98
ω6/ω3	12.4	13.0	12.8	0.21	0.55	0.94

Note ^1^: Treatment effect when *p* < 0.05, illustrated by different letters on the same line.

**Table 5:** Hematological parameters of lactating Jersey cows fed diets with or without free and protected protease.

Variables ^3^	Control	Free Protease	Protected Protease	SEM	*p*-Treat ^1^	*p*-Treat × Day ^2^	*p*-Period
WBC (10^3^/μL)	8.49 ^ab^	9.66 ^a^	7.41 ^b^	0.64	0.04	0.09	0.69
d1	9.92	9.51	8.99	0.69			
d14	7.98 ^b^	10.3 ^a^	6.86 ^b^	0.67			
d21	9.00	8.96	7.97	0.62			
Lymphocyte (10^3^/μL)	3.48 ^b^	5.53 ^a^	3.36 ^b^	0.46	0.05	0.03	0.54
d1	4.84	5.04	4.85	0.42			
d14	2.45 ^b^	6.10 ^a^	2.53 ^b^	0.47			
d21	4.52	4.97	4.20	0.44			
Granulocyte (10^3^/μL)	3.51 ^a^	3.47 ^a^	2.76 ^b^	0.22	0.05	0.17	0.87
Monocyte (10^3^/μL)	0.81 ^ab^	1.03 ^a^	0.67 ^b^	0.07	0.05	0.07	0.91
RBC (10^6^/μL)	5.17	4.98	4.98	0.13	0.89	0.93	0.95
Hemoglobin (g/dL)	9.50	9.29	9.38	0.25	0.92	0.95	0.97
Hematocrit %	26.7	26.3	26.5	0.67	0.95	0.96	0.96
Platelets (10^3^/μL)	362	463	411	24.0	0.11	0.06	0.89

Note ^1^: Treatment effect when *p* < 0.05, illustrated by different letters on the same line. Note ^2^: Interaction of Treatment × Day when *p* < 0.05, illustrated by different letters on the same line in each period. Note ^3^: In all groups, hematological variables are within the normal range for Jersey dairy cows.

**Table 6:** Clinical and metabolic biochemistry of lactating Jersey cows fed diets with or without free and protected protease.

Variables	Control	Free Protease	Protected Protease	SEM	*p*-Treat ^1^	*p*-Treat × Day ^2^	*p*-Period
Albumin (g/dL)	3.05	3.03	3.01	0.14	0.97	0.98	0.98
Cholesterol (mg/dL)	142	138	148	8.95	0.83	0.76	0.91
Creatinine (mg/dL)	0.46	0.46	0.46	0.03	0.99	0.98	0.96
Fructosamine (µmol/L)	309	279	299	15.82	0.65	0.51	0.79
GGT (U/L)	31.3	29.8	32.0	2.46	0.78	0.82	0.94
Glucose (mg/dL)	68.6	66.7	65.0	2.68	0.91	0.81	0.91
Total protein (g/dL)	6.01 ^b^	6.48 ^a^	6.33 ^ab^	0.19	0.05	0.12	0.90
AST (U/L)	94.3	89.6	82.3	6.50	0.66	0.59	0.85
Triglycerides (mg/dL)	9.21	8.95	8.02	0.53	0.52	0.35	0.92
Globulin (g/dL)	2.96 ^ab^	3.45 ^a^	2.79 ^b^	0.16	0.03	0.09	0.77
Urea (mg/dL)	33.7	35.4	35.6	1.71	0.05	0.01	0.66
d1	35.7	34.5	35.0	1.87			
d14	35.7	36.2	36.8	1.79			
d21	31.6 ^b^	34.5 ^a^	34.3 ^a^	1.63			

Note ^1^: Treatment effect when *p* < 0.05, illustrated by different letters on the same line. Note ^2^: Interaction of Treatment × Day when *p* < 0.05, illustrated by different letters on the same line in each period.

**Table 7:** Volatile fatty acids in the ruminal fluid of cows fed diets with or without free and protected protease.

Variables	Control	Free Protease	Protected Protease	SEM	*p*-Treat ^1^	*p*-Period
Acetic acid (mmol L^−1^)	43.6 ^c^	55.4 ^b^	62.8 ^a^	2.21	0.01	0.74
Propionic acid (mmol L^−1^)	13.6 ^b^	20.8 ^a^	19.4 ^a^	0.84	0.01	0.67
Isobutyric acid (mmol L^−1^)	0.64	0.66	0.66	0.03	0.96	0.93
Butyric acid (mmol L^−1^)	9.66 ^b^	13.40 ^a^	14.0 ^a^	0.58	0.01	0.65
Isovaleric acid (mmol L^−1^)	1.51	1.52	1.63	0.05	0.75	0.95
Valeric acid (mmol L^−1^)	1.42	1.30	1.38	0.06	0.87	0.88
Total VFA (mmol L^−1^)	70.5 ^b^	93.1 ^a^	99.9 ^a^	3.59	0.01	0.62
Number of protozoa (×10^5^ mL)	1.99 ^b^	2.76 ^a^	1.77 ^b^	0.25	0.01	0.83

Note ^1^: Treatment effect when *p* < 0.05, illustrated by different letters on the same line.

## Data Availability

Data is in the possession of the authors but may be made available upon request.

## Supplementary Materials

The following supporting information can be downloaded at: https://www.mdpi.com/article/10.3390/ani16121926/s1, Table S1. Feed and calculated composition of the basal diet of the cows used in this experiment. Table S2. Standardization of fatty acid measurements prior to analysis using ruminal fluid from cows fed free or protected protease; Figure S1: Image of the protected protease used in this experiment. The top image shows the protected protease immediately after its production, while the bottom image shows the dried protected protease, the form in which it was added to the concentrate and fed to the cows in this experiment.
