# Supplementary material for: Free and Protected Protease in the Diet of Lactating Jersey Cows: Effects on Performance, Milk Quality, Metabolism, Nutrient Digestibility, Microbiota, and Ruminal Environment
Source: Animals (Basel). 2026 Jun 22;16(12):1926. doi: 10.3390/ani16121926 (PMC13295378; doi:10.3390/ani16121926)
Supplement: Supplementary file 1 [file animals-16-01926-s001.zip › animals-4272391-supplementary.pdf]

Table S1. Feed and calculated composition of the basal diet of the cows used in this experiment.

**Feeds**	**% of dry matter**
Corn silage	47.6
Tifton hay	7.77
Ground corn	7.11
Corn gluten	3.55
Soybean meal	11.2
Wheat bran	1.98
Soybean hulls	2.66
DDGs	4.40
Dicalcemic phosphate	0.14
Calcitic limestone	0.54
Sodium chloride	0.25
Mineral and vitamin premix[1]	0.14
Sodium bicarbonate	0.54
Urea for livestock,	0.19
Pelleted feed to robot[2]	11.8
**Calculated chemical composition**	
Dry Matter	51.3
Crude protein	16.9
ADF	18.5
NDF	33.7
Starch	23.5
WSC	5.6
Ash	7.1
Lipids	2.36
Ca	0.51
P	0.37
Mg	0.21
K	1.11
Na	0.31
Cl	0.25
S	0.18
**Variables other**	
DCAD, mEq/kg	235
NEL, Mcal/kg	1.74
ME, Mcal/kg	2.64
MP, % DM	9.77
RDP, % DM	11.6
RUP, Base, % DM	5.3
Dig. RUP, % DM	4.2

Note 1: Commercial product (Cooper Alfa, Chapecó, Santa Catarina, Brazil).

Note 2: Robot feed (Cooper Alfa, Bom Jesus, Santa Catarina, Brazil).

Table S2. Standardization of fatty acid measurements prior to analysis using ruminal fluid from cows fed free or protected protease.

	Acetic acid	Propionic acid	Butyric acid	Isovaleric acid
R^2	0.9978	0.9973	0.9983	0.9983
Equation	y = 0.0171x + 0.0703	y = 0.0324x + 0.0198	y = 0.0436x + 0.005	y = 0.0541x + 0.0026
Linear range (mmol L^{-1})*	8.53 - 85.35	1.64 - 65.67	0.67 - 21.49	0.56 - 8.98
LOD (mmol L^{-1})	1.07	0.82	0.67	0.28
LOQ (mmol L^{-1})	8.53	1.64	0.67	0.56
Accuracy (%)	101.49	103.86	104.03	98.03
Repeatability (RSD)	4.53	4.90	3.50	3.49

* The linear range. LOD (limit of detection) and LOQ (limit of quantitation) were expressed in mmol of SFA for L of ruminal fluid

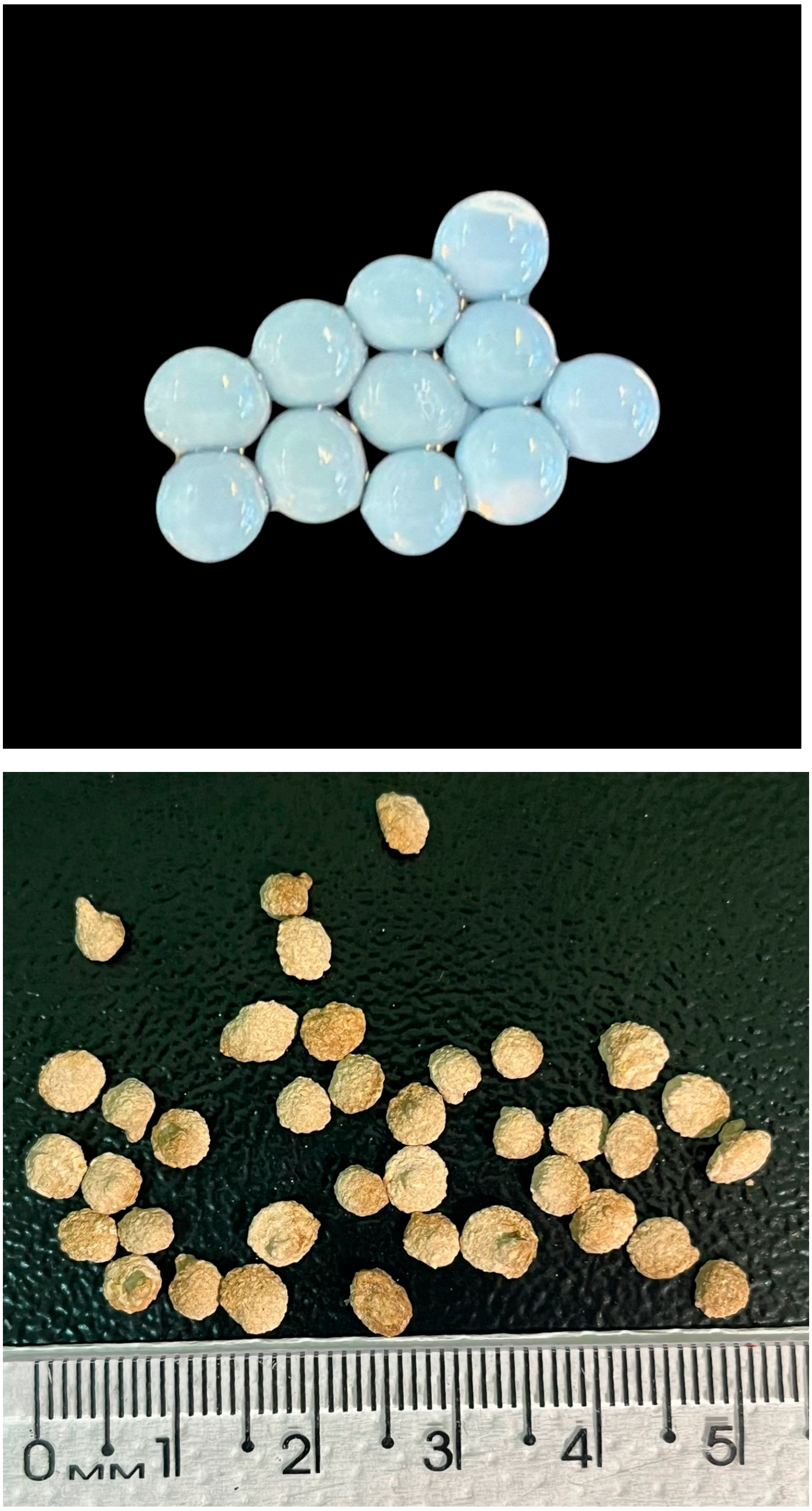

Figure S1: Image of the protected protease used in this experiment. The top image shows the protected protease immediately after its production, while the bottom image shows the dried protected protease, the form in which it was added to the concentrate and fed to the cows in this experiment.
